# Development and Content Validity of Cervicogenic Headache Patient Questionnaire: New Tool for Assessing Severity and Impact

**DOI:** 10.7759/cureus.68432

**Published:** 2024-09-02

**Authors:** Pallavi Harjpal, Moh'd Irshad Qureshi, Manali S Chitlange, Vaibhav P Anjankar, Rakesh Krishna Kovela, Pratik Phansopkar

**Affiliations:** 1 Department of Neuro-Physiotherapy, Ravi Nair Physiotherapy College, Datta Meghe Institute of Higher Education and Research, Wardha, IND; 2 Department of Anatomy, School of Higher Education and Research, Jawaharlal Nehru Medical College, Datta Meghe Institute of Higher Education and Research, Wardha, IND; 3 Department of Physiotherapy, Nitte Institute of Physiotherapy, NITTE (Deemed to be University), Mangaluru, IND; 4 Department of Musculoskeletal Physiotherapy, Ravi Nair Physiotherapy College, Datta Meghe Institute of Higher Education and Research (DU), Wardha, IND

**Keywords:** headache, content validity, scale development, patient questionnaire, cervicogenic headache

## Abstract

Background and aim: Cervicogenic headache (CGH) is a complicated and common headache disorder that may present itself with cervical spine dysfunction, irritation of the nerves, central sensitization, and muscular tension. Theoretically, this diagnosis should be an exclusionary diagnosis, considering the fact that it requires extended clinical assessment of the cervical spine and an evaluation for other primary headache types. CGH represents a clinical challenge as they habitually present with an array of nonspecific manifestations highly variable among patients. Management of CGHs is properly based on accurate diagnosis and sound understanding of the complaints of the individual. The development of CGH still carries with it a palpable gap in meaningful literature related to really effective assessment tools for the condition. The aim of the current study was to develop and investigate the validity of the content of the Cervicogenic Headache Patient Questionnaire. This questionnaire is designed especially to measure pain intensity, its impact on daily activities, disturbance in sleep, and the overall quality of life in subjects with CGHs.

Methodology and results: The content validity ratio (CVR) and Content Validity Index (CVI) have been used to thoroughly examine the content validity. Each item was rated by 10 experts for relevance and clarity. The scale consists of two main parts: demographic information and symptoms. Under the demographic section, the patient is asked for their age, sex, occupation, and any relevant medical history. The Symptom Checklist contains 10 questions concerning headache frequency, intensity, duration, location, and contributing factors, as well as how headaches affect daily activities, sleep, and quality of life. Item-CVI scores ranged from 0.60 to 1.00, and Scale-CVI/Ave was 0.95, which indicates strong overall content validity. The Scale-CVI/Universal Agreement was 0.83, meaning that most items are of high relevance. The clarity assessments resulted in I-CVI ratings of 1.00 for the majority of items. Using CVR analysis, items 1, 2, 5, 6, 7, and 10 all had a CVR of 1.00, whereas all the rest ranged from 0.40 to 0.80, suggesting unanimous agreement among the experts.

Conclusion: The results underline the strength of the questionnaire in covering all the critical dimensions of cervicogenic headaches, such as pain, daily functioning, sleep, and quality of life. The scores provided by experts for content validity and clarity were high; hence, it is suitable for use as a comprehensive tool both in clinical and research applications.

## Introduction

Cervicogenic headache (CGH) is a chronic headache that originates from the neck or upper cervical spine and can refer to the head and face. It is a secondary headache occurring as a consequence of pathology in the cervical spine. It is often associated with reduced neck mobility, muscle tenderness, and poor posture [1]. Pain is generally first perceived in the neck and then radiates to the head or face. The prevalence of CGH in patients suffering from headache disorders is considered to be around 15-20% in clinical practice [2]. This is more common in adults than children, although prevalence varies by diagnostic criteria and population studied. It is often associated with chronic neck pain and tends to be more common among people of professions or activities involving repetitive neck strain, poor posture, and trauma [3]. For instance, research shows that the general population typically has a prevalence of about 2-4% of CGH. At the same time, in some clinical groups, especially those with chronic musculoskeletal conditions or occupational risk factors, it is much higher [4]. The pathophysiology of CGH includes a complex interplay between cervical spine pathology and the central pain processing mechanisms [5]. Fundamentally, there are only a few mechanisms underlying the development of CGH which are cervical spine dysfunction, nerve irritation and referred pain, central sensitization, muscle tension, and postural problems [6]. It requires knowledge regarding prevalence and pathophysiology for diagnosis and successful management [7]. CGH is a clinical challenge, as they usually present with an array of nonspecific manifestations that vary a lot among patients. Proper management of CGHs is based on accurate diagnosis and sound understanding of the complaints of the individual [8].

Despite the developments in understanding CGH, there is a palpable gap in meaningful literature related to effective assessment tools regarding the condition. The present assessment methods are based mostly on clinical assessment or subjective patient history, often non-standardized and insensitive; hence, diagnosis and management are variable [9]. Indeed, traditional assessments in most cases fail to provide a comprehensive estimation of the effects of CGH on patients' daily functioning and quality of life, missing crucial information about relevant functional impairments. This supports the need for patient-reported outcome measures (PROM) to demonstrate symptomatology at a greater level of detail and nuance from the patient's perspective to increase diagnostic accuracy and will enhance treatment evaluation by monitoring changes in symptoms and functional status over time. The inclusion of PROMs can be made to take account of the current assessment deficiencies and to follow up on the recent trend toward more patient-centered care so that treatment plans are more specifically tailored to individual needs [7].

One of the most common tools to measure health-related changes due to health-management interventions is patient-reported outcome measures [10]. When developing these instruments, several important properties need to be taken into consideration, such as reliability and validity. On the list of the most distinguishing properties appears content validity. Content validity reflects the extent to which a measure or questionnaire genuinely represents what one is seeking to measure [11]. Content validity is seen as an important measurement property by the COSMIN (Consensus-based Standards for the Selection of Health Measurement Instruments) initiative [12]. It should ensure that PROMs are not only rigorously scientific but also pertinent and representative of patient experience and outcomes [13]. Evidence from earlier studies indicated that appropriate content validity testing would enhance the reliability and relevance of diagnostic measures [14,15]. Different headache types have a number of outcome measures available to assess them, but specifically for CGHs, no special tool has been devised to evaluate all aspects. What is needed is a valid measure that can detail physical pain and daily activity, sleep disturbance, and quality of life, parallel checking now and then regarding the psychometric properties, and use of scale measures in treatment outcomes in the clinical set-up. This study intends to construct a scale that can evaluate these domains comprehensively in CGH subjects. The major objective is to generate and define the items together with the domains of a scale by preparing the scale and establishing content validity using the Delphi Method.

## Materials and methods

**Figure 1:**
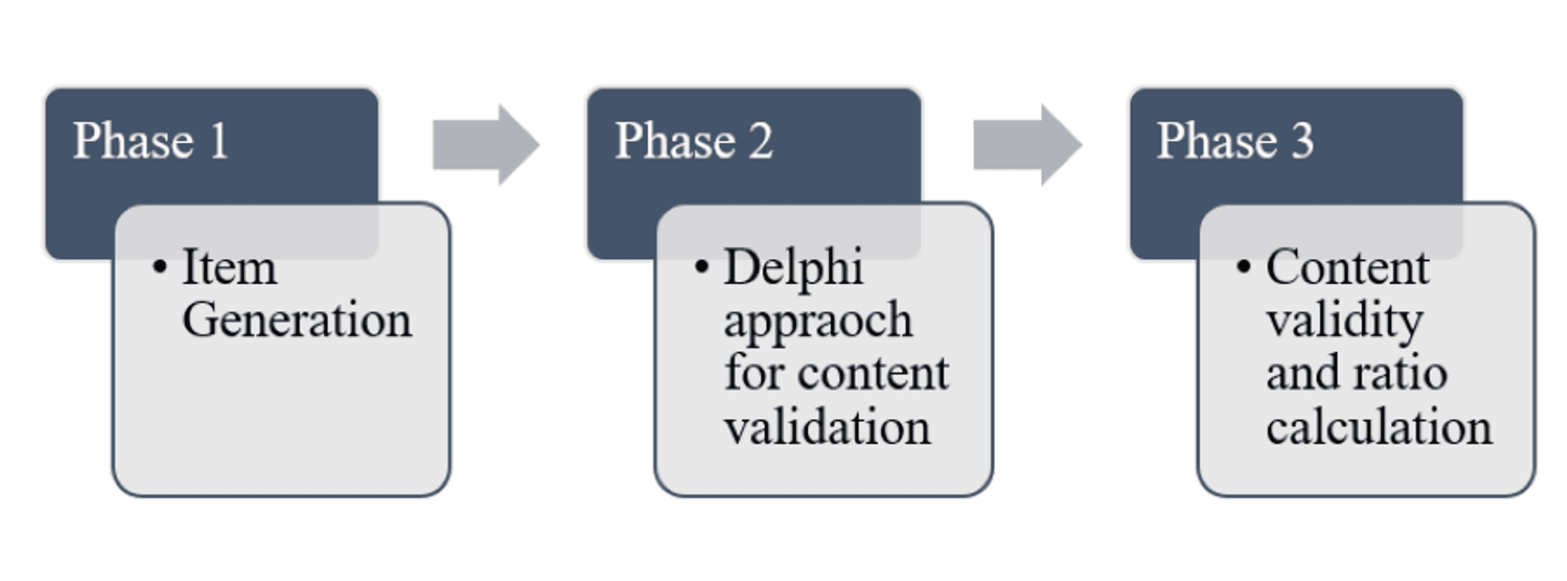
Phases of the study

This study was undertaken at Datta Meghe Institute of Higher Education and Research, Wardha, Maharashtra, India. It included three major steps: first, generating items to assess the impact of CGH on the quality of life of patients based on a careful review of existing literature and study item suggestions by patients and experts in relevant fields through interviews; secondly, the items were evaluated for content validity using the Delphi Method. The third phase was to compute the content validity index (CVI) and the ratio [15]. Figure 1 depicts these phases.

The first phase was the item generation phase where systematic development of domains and items was done. It was followed by content validation through the Delphi Method and content validity calculation. A Pilot study was also conducted for the same. These phases are depicted in Table 1.

**Table 1:** Various phases of the study CGH: cervicogenic headache; I-CVI: item-level content validity index; S-CVI/UA: scale-level content validity index/universal agreement; S-CVI/Ave: scale-level content validity index/Average method

Phase 1	
Systematic Development of Domains and Items	This phase would consist of generating items concerning domains of physical pain, impact on activities of daily living, sleep disturbances, and, quality of life. It has been further divisible into the following three sub-phases: 1. Extensive literature review: The PubMed database was scouted from 2004 to 2024 using terms like CGH, quality of life, headache, and CGH questionnaires. 2. Expert interviews: Two expert physiotherapists with more than five years of experience conducted in-depth interviews to generate relevant domains and associated items. 3. Patient Interviews: Face-to-face interviews were conducted with patients who have a CGH condition to know their problems and difficulties related to CGH in their health.
Phase 2	
Content Validation through the Delphi Method	The scale was shared online with a panel of 10 experts (Physiotherapist, Occupational Therapist, Neurologist, Orthopedician, Pain specialist) working in the domain of CGH in India, each holding a master's degree or higher and having experience of at least five years with CGH patients. They were invited via email to participate in the content validation using a Google form [16]. They were provided with the information of scale and the need for the development and content validation of the same. The items were rated for relevance and clarity by the experts on a four-point scale: 4=highly relevant and clear, 3=quite relevant, 2=somewhat relevant, and 1=not relevant and, for clarity, very clear (4), clear but needs minor revision (3), item needs some revision (2), and not clear (1). The careful consideration of the response decided whether items were relevant to be included in the questionnaire. The inclusion/exclusion of items was based on the available literature on the CGH. There were two rounds of the Delphi process that were conducted.
Phase 3	
Calculation of Content Validity Index	The table shows the item-level content validity index (I-CVI) and the scale-level content validity index, computed by both methods, the Universal Agreement method (S-CVI/UA) and the Average method (S-CVI/Ave) [14,17]. The threshold for acceptance, according to Lynn MR, for an S-CVI of at least 0.78 with 6-10 experts and S-CVI/Ave of 0.90, is excellent [13,18]. The content validity ratio was also computed [19].
Pilot Testing	Following the expert review, 24 patients were put through a pilot test for clarity, perception of the scale, and its practicality [20]. The patients provided informed consent, completed the scale, and then provided feedback and opinions about items and responses. The feedback was used to ascertain problems and assess the practicality of the questionnaire.

Cervicogenic Headache Patient Questionnaire (CHPQ)

The CHPQ is a research instrument that is used to assess the impact of CGHs on the patient’s quality of life. It consists of two main parts: demographic information and symptoms. Under the demographic section, the patient is asked for their age, sex, occupation, and any relevant medical history. The Symptom Checklist contains 10 questions concerning headache frequency, intensity, duration, location, and contributing factors, as well as how headaches affect daily activities, sleep, and quality of life. Some of the questions are open-ended, while others are closed-ended in the Likert scale format and on a visual analog scale. It is a self-administered tool answered by the patient and takes about 15 minutes to complete.

The data that will be collected from the questionnaire will provide information on the severity and level of disability of cervicogenic headache and will aid in the identification of factors that influence the quality of life among patients with a disability. This questionnaire will facilitate monitoring of the progress and effectiveness of treatment interventions concerning cervicogenic headache. This questionnaire is based on the literature and clinical guidelines on cervicogenic headache and based on experts’ and patients’ feedback. Interpretation will be as follows: 01-10: Mild impact, 11-20: Moderate impact, 21-30: Severe impact, 31-40: Extremely severe impact.

Data analysis

Descriptive statistics were used to present demographic data. Items on the “Cervicogenic Headache Patient Questionnaire” were rated for relevance and clarity according to a four-point scale: For relevance, Not Relevant (1), Somewhat Relevant (2), Quite Relevant (3), and Highly Relevant (4) and, for clarity, very clear (4), clear but needs minor revision (3), item needs some revision (2), and not clear (1) with a panel of 10 experts, in view of content validity. In this regard, each item was computed for a CVI by approximating the proportion of targeted experts rating an item as either “Quite Relevant” or “Highly Relevant.”

For each item, the I-CVI was computed as the proportion of experts rating an item as either 3 or 4, divided by the total number of raters to obtain a value between 0 and 1 [14]. The S-CVI was obtained by averaging all I-CVIs. It could be calculated for modified kappa with Pc defined as the probability of chance agreement for each item according to the formula: Pc = [N! / A! (N-A)!] * 0.5^N, where N denotes the total number of raters and A is the number of raters who found the item relevant [21]. Kappa was computed according to the formula: K = (I-CVI - Pc) / (1 - Pc), where Pc is the probability of chance agreement and I-CVI represents the item-CVI for each item. The scores were then interpreted according to the threshold set as follows: scores from 0 to 0.4 as “Poor,” from 0.4 to 0.59 as “Fair,” from 0.6 to 0.74 as “Good,” and from 0.75 to 1 as “Excellent” [22]. After completion of the Delphi process, the scale developed has been copyrighted and registered with the Government of India. The registration number is L-145482/2024.

## Results

In the current study, the content validity of the “Cervicogenic Headache Patient Questionnaire” was checked by the CVI, with ratings taken from a panel of 10 experts. Each expert rated item relevance and clarity for the questionnaire on a scale from 1-4. Proportions by experts who agreed about the relevance of an item were computed; the consequent item-CVI scores varied from 0.60 to 1.00. Items with item-level content validity index (I-CVI) scores of 1.00 were excellent, showing unanimous agreement about the relevance of these items. Questions 6 and 8 had respective I-CVI scores of 0.60 and 0.80, indicating fair to excellent agreement. The scale-level content validity index based on the average method (S-CVI/Ave) was 0.95, which is the average of I-CVI across all items and indicates high overall content validity. Additionally, the S-CVI/UA, which gives the proportion of items at or above the acceptable threshold of relevance, was 0.83. This implies that most items on the questionnaire are of high significance to the measured construct and that there are a few items that need revision. In general, this content validity analysis suggests that the instrument is very sound and that the items on it generally received excellent relevance ratings.

For clarity assessment, the majority of the questions in the questionnaire are of a high order of excellence. Items 1-5, which solicited information about the patients themselves, their medical history, the frequency of headaches per week and per day, and the duration of a headache, all scored with excellent clarity. Each question took a perfect I-CVI rating of 1.00 and a proportion of agreement (pc) rating of 0.00, indicating that they are very clear and precisely understood by all evaluators. Similarly, items 7, 8, 9, 10, 11, and 12, which elicit information regarding pain intensity, headache triggers, relief measures taken, impact of the headache in daily life, sleep disturbances, and quality of life in general, were also rated for their clarity. These questions retained a perfect I-CVI of 1.00 and a pc of 0.00, showing a very strong consensus regarding clarity among experts. Importantly, the clarity of Question 6, which asks about pain location, was rated as excellent with a perfect I-CVI of 1.00 and proportion of agreement pc = 0.00, indicating that it is very clear to all evaluators. On the whole, clarity has still remained very high for this questionnaire, with all its questions rated as excellent. The CVI is shown in Table 2.

**Table 2:** Content Validity Index I-CVI: item-level content validity index; pc: proportion of agreement; k*: modified kappa; S-CVI/Ave: scale-level content validity index based on the average method

	Relevance				Clarity				
Questions	Agreement	I-CVI	pc	k*	Agreement	I-CVI	pc	k*	Interpretation
Question -1 (Personal Information)	10/10	1.00	0.00	1.00	10/10	1.00	0.00	1.00	Excellent
Question -2 (Medical History)	10/10	1.00	0.00	1.00	10/10	1.00	0.00	1.00	Excellent
Question -3 (Headache Frequency/week)	10/10	1.00	0.00	1.00	10/10	1.00	0.00	1.00	Excellent
Question -4 (Headache Frequency/day)	10/10	1.00	0.00	1.00	10/10	1.00	0.00	1.00	Excellent
Question -5 (Headache Duration)	10/10	1.00	0.00	1.00	10/10	1.00	0.00	1.00	Excellent
Question -6 (Pain Location)	6/10	0.60	0.21	0.50	6/10	1.00	0.00	1.00	Fair
Question -7 (Pain Intensity)	10/10	1.00	0.00	1.00	10/10	1.00	0.00	1.00	Excellent
Question -8 (Triggers)	8/10	0.80	0.04	0.79	8/10	1.00	0.00	1.00	Excellent
Question -9 (Relief Measures)	10/10	1.00	0.00	1.00	10/10	1.00	0.00	1.00	Excellent
Question -10 (Impact on Daily Life)	10/10	1.00	0.00	1.00	10/10	1.00	0.00	1.00	Excellent
Question -11 (Sleep Disturbance)	10/10	1.00	0.00	1.00	10/10	1.00	0.00	1.00	Excellent
Question -12 (Overall Quality of Life)	10/10	1.00	0.00	1.00	10/10	1.00	0.00	1.00	Excellent
Proportion Relevance		0.95				1			
		S-CVI/Ave				S-CVI/Ave			

The content validity ratio (CVR) of the “Cervicogenic Headache Patient Questionnaire” was computed using the Lawshe method. Each item in the measure was rated by 10 experts ticking either column “Relevant” or “Not Relevant.” The CVR for each item was computed using the formula: CVR = (ne - N/2) / (N/2), where ne is the number of experts who rated the item as essential, and N is the total number of experts [19]. The results for each item were as follows: Questions 1, 2, 5, 6, 7, 10: All returned a CVR of 1.00, representing unanimous agreement on the relevance of the questions. Questions 3 and 11: Returned CVRs of 0.60, indicating moderate agreement about their relevance. Questions 4 and 9: Both were 0.80, thereby showing strong consensus about their relevance. Question 8: It was 0.40, the experts being less convinced of its relevance. Question 12: It was 0.40, the experts being less convinced of its relevance.

The average CVR across all the items was 0.80. There is an extremely high level of agreement among the experts regarding the relevance of the items. This analysis lends support to the content validity of the questionnaire and confirms that most items are considered essential by the expert panel. The content validity ratio (CVR) calculation is shown in Table 2.

**Table 3:** Content Validity Ratio

Questions	Agreement	Content Validity Ratio
Question -1 (Personal Information)	10/10	1.00
Question -2 (Medical History)	10/10	1.00
Question -3 (Headache Frequency/week)	8/10	0.60
Question -4 (Headache Frequency/day)	9/10	0.80
Question -5 (Headache Duration)	10/10	1.00
Question -6 (Pain Location)	10/10	1.00
Question -7 (Pain Intensity)	10/10	1.00
Question -8 (Triggers)	7/10	0.40
Question -9 (Relief Measures)	9/10	0.80
Question -10 (Impact on Daily Life)	10/10	1.00
Question -11 (Sleep Disturbance)	8/10	0.60
Question -12 (Overall Quality of Life)	7/10	0.40
	Average	0.80

## Discussion

The present study was undertaken to develop a comprehensive scale for the assessment of critical domains in patients with cervicogenic headache, specifically related to physical pain, influence on everyday activities, sleep disturbances, and quality of life in general. The Cervicogenic Headache Patient Questionnaire was developed in response to the recognized need for a valid measure to cover the complexity of CGH. It is often noted that most headache measures usually fail to capture the full spectrum of symptoms of CGH and their related functional impairment. The CHPQ is based on items of physical pain, limitations in daily activities, sleep problems, and quality of life. In essence, it captures a broad view of patient experiences and treatment outcomes.

Content validity is important in ensuring the scale adequately represents what it measures in validating the content of the CHPQ, the Delphi Method was employed by seeking expert feedback in order to refine the items in the questionnaire and confirm them to be relevant and comprehensive. This stringent approach will ensure that the scale is extensive enough to cover the core aspects of CGH whose information lies within the experience of the patients. Using the CHPQ, hence, one can assess physical pain in terms of its intensity, frequency of occurrence, and its consequence on everyday life [23]. This focus is essential since pain is a primary symptom of CGH, which has a significant influence on the quality of life of the patients. With the itemization of detailed assessment items on pain, the CHPQ may yield crucial information regarding the variation of physical pain over time and treatment. Another critical area that the CHPQ covers is the interference of CGH in daily activities [6]. Although previous studies have demonstrated that CGH can certainly produce impairments in daily functioning, such as work, leisure, or home activities, the CHPQ tries to record such limitations at some length, offering a clearer idea regarding the way CGH affects the performance of daily tasks and normal activities of patients [24].

Patients frequently complain about sleep in relation to CGH, which might even aggravate the situation in some cases [4]. The CHPQ satisfies this key facet of patient well-being through seeking sleep-related items. This enables investigation into fuller descriptions of the secondary impact of CGH and may also support targeted interventions for sleep quality improvement paired with headache management. Incorporation of quality-of-life measures into the CHPQ is therefore important for understanding the broader effects of CGH on the general well-being of patients [25]. Physical, emotional, and social dimensions of quality of life are all affected negatively by chronic headache disorders [26]. The quality of life, focused on by the CHPQ, aids in the assessment of the general impact of CGH and the effectiveness of interventions on patient life satisfaction.

Using the CVI and expert feedback from 10 sources, the content validity of the “Cervicogenic Headache Patient Questionnaire” was thoroughly assessed. For all questions, CVI values were all between 0.60 and 1.00, which is a good range for relevance. The scores for items 6 and 8 were 0.60 and 0.80, respectively. The overall content validity was demonstrated by the scale-level content validity index based on the average method (S-CVI/Ave) of 0.95, and the scale-level content validity index based on the Universal Agreement method (S-CVI/UA) of 0.83 suggested that most of the items were very relevant, albeit some might need to be revised [18]. The evaluations of clarity were quite good. Items 7-12, which deal with pain severity and issues caused by it, and items 1-5, which ask about the patient's demographic information and headache details, all got flawless I-CVI ratings of 1.00 and pc of 0.00, demonstrating their clarity. CVR was calculated by using the Lawshe method [27]. Item analysis has been found to have expert consensus for the relevance on Questions 1, 2, 5, 6, 7, and 10, since each of them has a calculated CVR value of 1.00. However, the level of agreement has been found between experts for Questions 3 (CVR = 0.60) and 11 (CVR = 0.60) for presence while for Questions 4 (CVR = 0.80) and 9 (CVR = 0.80). This result tests high content validity with a mean CVR of 0.80, which indicates that most of the items were rated as essential by the expert panel.

Limitations

While the content validity study of the Cervicogenic Headache Patient Questionnaire is itself informative regarding the instrument’s relevance and comprehensiveness, there are a few limitations. The assessment in this study of content validity relied on experts’ reviews - important but intrinsically subjective and likely to be influenced by experts' varying perspectives and expertise. Again, the absence of an empirical measurement of reliability decreases confidence about whether the resulting questionnaire will produce consistent results in different populations and contexts. In order to address these concerns and provide evidence of the robustness of the questionnaire, further research is planned for its reliability assessment.

Further recommendations

To ensure the Cervicogenic Headache Patient Questionnaire (CHPQ) is widely applicable among various conditions where cervicogenic headache is secondary to some primary pathology and to assess its sensitivity to various patient demographics, it is crucial to expand testing to include diverse age groups, ethnicities, and individuals with different comorbid conditions. This comprehensive approach will help validate the questionnaire's effectiveness across a broad spectrum of populations. Moreover, a comparison of the CHPQ with other established headache assessment tools will yield useful information on its relative effectiveness and point out its shortcomings. Clinical trials, in which the CHPQ has been included, have also more fully tested its use as a tool to measure treatment outcomes and patient responses to interventions for cervicogenic headache. Again, it will be important that health providers are given training and other resources for the best use of the CHPQ in diagnosis and treatment planning to realize optimum clinical effect. In addition, its accessibility, use, and data collection both for patients and clinicians might further be improved by considering its digital or app-based versions.

## Conclusions

Development and content validation of the CHPQ has been an important step toward standardization in the assessment of cervicogenic headaches. Relating to core domains like pain, limitations in daily activities, sleep-related problems, and quality of life, the CHPQ is a multidimensional tool that can be used in both clinical practice and research. Further studies should continue to examine the psychometric properties of this scale and its application in various clinical settings to establish its effectiveness and reliability.
